# Supplementary material for: MicroRNA-375 Is Induced during Astrocyte-to-Neuron Reprogramming and Promotes Survival of Reprogrammed Neurons when Overexpressed
Source: Cells. 2023 Sep 3;12(17):2202. doi: 10.3390/cells12172202 (PMC10486704; doi:10.3390/cells12172202)
Supplement: Supplementary file 1 [file cells-12-02202-s001.zip › cells-2551045-supp layout.pdf]

MicroRNA-375 is Induced during Astrocyte-to-Neuron Reprogramming and Promotes Survival of Reprogrammed Neurons when Overexpressed

Xuanyu Chen [1,*], Ivan Sokirniy [2], Xin Wang [3], Mei Jiang [1], Natalie Mseis-Jackson [1], Christine Williams [4], Kristopher Mayes [1], Na Jiang [1], Brendan Puls [3], Quansheng Du [1], Yang Shi [1,5] and Hedong Li [1,*]

[1] Department of Neuroscience & Regenerative Medicine, Medical College of Georgia at Augusta University, Augusta, GA 30912, USA; mejiang@augusta.edu (M.J.); nmseis@augusta.edu (N.M. -J.); kmayes@augusta.edu (K.M.); njiang@augusta.edu (N.J.); qdu@augusta.edu (Q.D.); yshi@augusta.edu (Y.S.)

[2] Department of Biomedical Engineering, The Pennsylvania State University, University Park, PA 16802, USA; iys5072@psu.edu

[3] Department of Biology, The Pennsylvania State University, University Park, PA 16802, USA; xin.wang@pennmedicine.upenn.edu (X.W.); bpuls@hitechinstruments.com (B.P.)

[4] Department of Chemistry & Biochemistry, College of Science & Mathematics, Augusta University, Augusta, GA 30912, USA; chwilliams4@augusta.edu

[5] Division of Biostatistics and Data Science, Department of Population Health Sciences, Medical College of Georgia at Augusta University, Augusta, GA 30912, USA

* Correspondence: xuachen@augusta.edu (X.C.); hedli@augusta.edu (H.L.); Tel.: +1 706-667-4826 (H.L.)

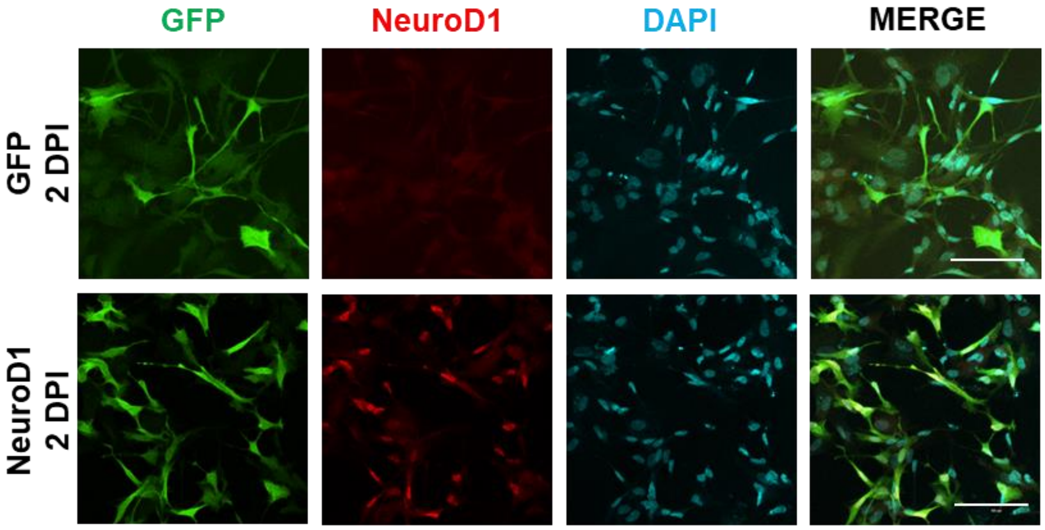

Figure S1. Retro-NeuroD1-GFP increased the nuclear NeuroD1 (ND1) expression in HA cells. At 2 days post infection (DPI), HA cells infected with retro-GFP or retro-NeuroD1-GFP were fixed and stained with anti-GFP, anti-NeuroD1, and DAPI. Scale bars, 100 μm. Related with Figure 1 (d).

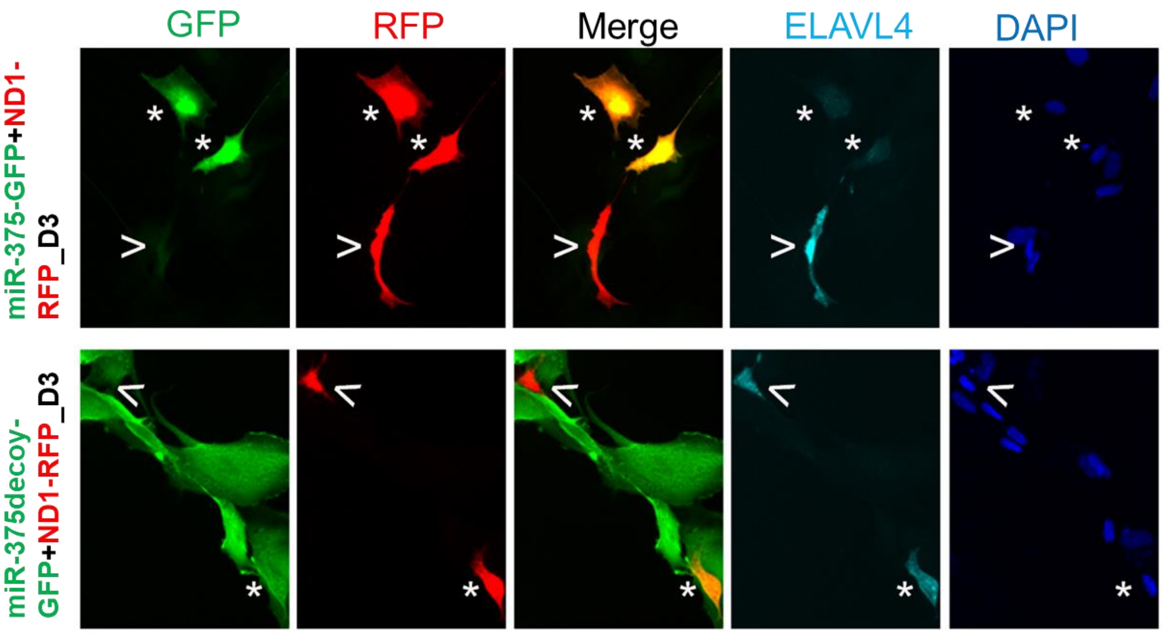

Figure S2. MiR-375 regulates ELAVL4 expression at D3 of NeuroD1-mediated AtN reprogramming. >, ND1 alone-infected HA; *, coinfected HA. Related with Figure 4(g).

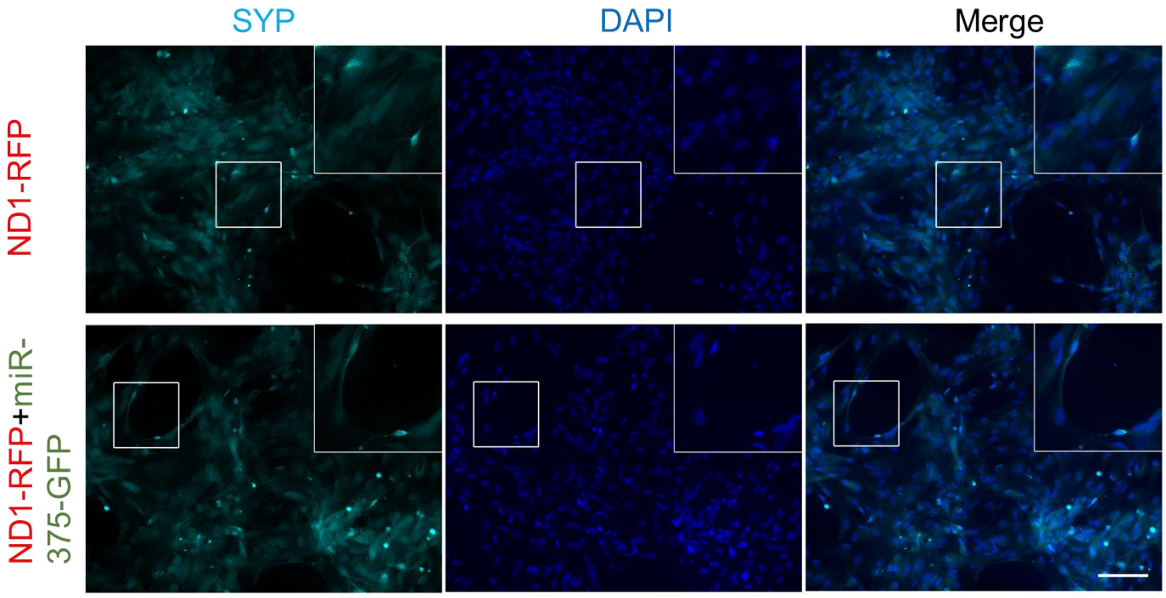

Figure S3. Representative immunostaining images of synaptophysin (SYP) in long-term cultures of FACS-sorted cells infected with retro-ND1-RFP or ND1-RFP+miR-375-GFP at 30 DPI. Scale bar, 100 μm. Related with Figure 9(c).

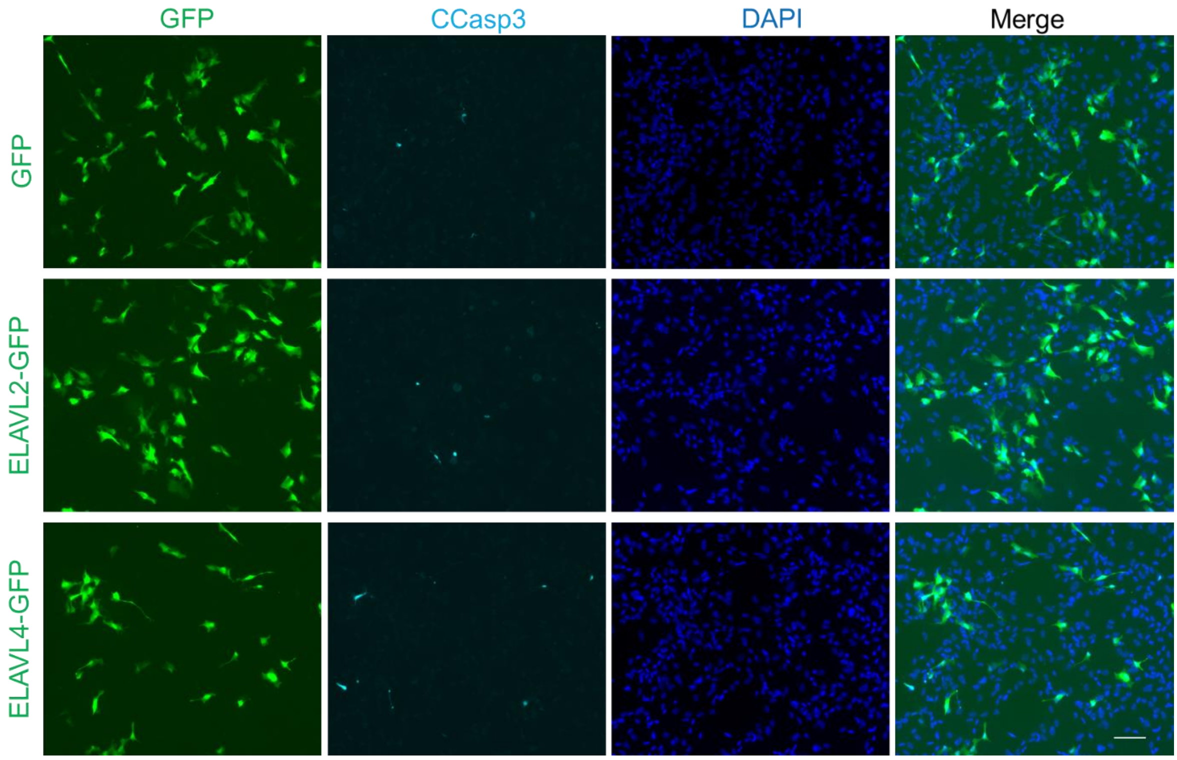

Figure S4. Representative immunostaining images of HA infected with retro-GFP, retro-ELAVL2-GFP, or retro-ELAVL4-GFP. Scale bar, 100 µm. Related with Figure 10(d).

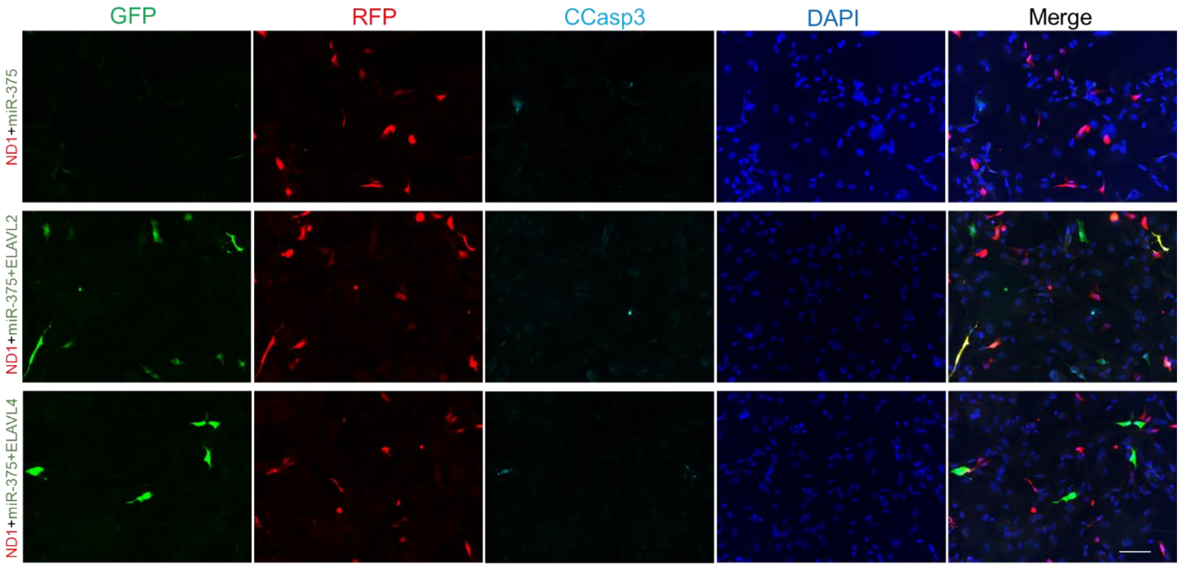

Figure S5. Representative immunostaining images of HA coinfected with retro-ND1-RFP+miR-375-GFP, retro-ND1-RFP+miR-375-GFP+ELAVL2-GFP, or retro-ND1-RFP+miR-375-GFP+ELAVL4-GFP. Scale bar, 100 µm. Related with Figure 10(e).
